# Ensuring Privacy When Integrating Patient-Based Datasets: New Methods and Developments in Record Linkage

**DOI:** 10.3389/fpubh.2017.00034

**Published:** 2017-03-02

**Authors:** Adrian P. Brown, Anna M. Ferrante, Sean M. Randall, James H. Boyd, James B. Semmens

**Affiliations:** ^1^Centre for Population Health Research, Curtin University, Bentley, WA, Australia

**Keywords:** record linkage, data integration, privacy, encryption, data quality, linkage quality

## Abstract

In an era where the volume of structured and unstructured digital data has exploded, there has been an enormous growth in the creation of data about individuals that can be used for understanding and treating disease. Joining these records together at an individual level provides a complete picture of a patient’s interaction with health services and allows better assessment of patient outcomes and effectiveness of treatment and services. Record linkage techniques provide an efficient and cost-effective method to bring individual records together as patient profiles. These linkage procedures bring their own challenges, especially relating to the protection of privacy. The development and implementation of record linkage systems that do not require the release of personal information can reduce the risks associated with record linkage and overcome legal barriers to data sharing. Current conceptual and experimental privacy-preserving record linkage (PPRL) models show promise in addressing data integration challenges. Enhancing and operationalizing PPRL protocols can help address the dilemma faced by some custodians between using data to improve quality of life and dealing with the ethical, legal, and administrative issues associated with protecting an individual’s privacy. These methods can reduce the risk to privacy, as they do not require personally identifying information to be shared. PPRL methods can improve the delivery of record linkage services to the health and broader research community.

## Introduction

Unabating growth in the creation of data, coupled with advances in information technology and Internet connectivity, provides tremendous potential for data-driven breakthroughs in the understanding, treatment, and prevention of disease. These health research innovations are being complemented by data from non-traditional sources (i.e., from sources other than administrative health and survey records). Opportunities include the use of mobile phone records (1) and Google search histories (2) for disease surveillance, patient collected data from wearable devices (3), and manual journaling through mobile phone applications (4). Data from the private health sector and government administrative datasets that lie outside the health sector (5) are also of interest, as is spatial information that has direct application for understanding exposures and inequalities (6). Genetic information unavailable a generation ago is already used in clinical decision making (7), and its importance is only likely to increase. The key to unlocking these data is in relating details at an individual patient level to provide an understanding of risk factors and appropriate interventions (8).

A key methodology that has supported health research is record linkage, a process of accurately bringing together records from multiple datasets that belong to the same person. Through record linkage, it has been possible to construct and analyze population-wide datasets comprising “linked” administrative records pertaining to each individual. Health-based record linkage frameworks have been established, which routinely integrate data from hospital admissions, emergency departments, primary care facilities, birth, death, and disease registries (1, 2), creating a rich analytic resource to support evidence-based decision making (9–11).

Present models of record linkage use trusted third parties (TTPs) or data linkage units (DLUs) to accurately match records using personal identifiers (12). Incorporating information from new and diverse data sources into these linkage frameworks are likely to have significant benefits to research; however, the operational and administrative overheads are substantial. Technical issues (i.e., scalability, efficiency) and effects on linkage quality (accuracy) will also be impacted and need to be assessed.

Sharing of public and private datasets also presents privacy and confidentiality challenges. Protecting the privacy of individuals is paramount in the record linkage process and essential to maintain community support and trust. There are serious ethical implications in combining information on individuals (generally without direct consent) from government and other sources; essentially a form of surveillance of an entire population. For some privacy advocates, this is a bridge too far, conjuring up images of an Orwellian dystopia or the excesses of totalitarian regimes (13, 14). Health researchers argue that privacy risks can be minimized and that the public benefit of utilizing these rich datasets outweighs the risk to privacy; that is, there is an ethical imperative to conduct record linkage for research (15). The public’s view on this issue is not always clear; numerous surveys have been conducted in Australia, which sometimes return contradictory results regarding Australian views on the use of personal health information [see Ref. (16). for a review]. Similar contradictions have been observed in results from Canadian surveys (14).

While a number of existing processes and techniques are used to maintain patient privacy during record linkage (17), the development of new and improved linkage methods may provide an opportunity for alternative approaches that further reduce privacy risks without compromising on linkage quality.

This article discusses the emergence and potential benefit of record linkage techniques that limit the release of personal identifiers for linkage. These methods, collectively referred to as privacy-preserving record linkage (PPRL), operate in such a way that they do *not* require the release of personally identifying information by data custodians. PPRL methods work on information that has been permanently encoded, encrypted, or transformed before releasing the data for linkage. Through PPRL methods, the benefits of linkage can be realized without the risks associated with disclosure of personal information.

## Existing Record Linkage Frameworks

There is a long history in Australia of record linkage supporting both jurisdictional level and national research and health decision making (10, 12, 18). Record linkage capabilities in all jurisdictions (19–21) have recently been strengthened, and in many cases expanded, through strategic national investment: through the National Collaborative Research Infrastructure Strategy in Australia; the Canadian Institutes of Health Research in Canada; and through the Farr Institute initiative in the United Kingdom (22).

The record linkage framework adopted by most of these jurisdictions is a TTP model, whereby dedicated linkage units undertake record linkage to service and support research. Administrative data collections (such as hospital discharges, emergency presentations, mortality, and cancer registers) have typically formed the backbone of enduring record linkage systems (18, 23). Such collections are highly confidential, containing sensitive personal information that is protected by law.

## Record Linkage and Privacy

Linkage of person-level records through the use of personally identifying information, and generally without consent, has significant ethical and legal implications that have been at the forefront of issues confronted and addressed by DLUs (12, 24).

The extent to which data can be used in record linkage depends on the applicable legislation in each jurisdiction. Some administrative collections are bound by specific laws which either prohibit or severely curtail the release of personal information from these systems.^1^ It has been claimed that more than 500 secrecy and privacy provisions exist in Australian Commonwealth laws, imposing considerable limits on the availability and use of identifiable data (25). At Commonwealth level, privacy laws permit some level of disclosure of personal information by authorities for human research (*Commonwealth Privacy Act 1988 s 95*). The release of personal data for linkage can be authorized if public benefit outweighs the privacy of individuals (26).

Working within these legal frameworks, data custodians, DLUs, and the research community in Australia have developed secure data access and usage models that provide important safeguards to privacy. DLUs have also implemented best practice data governance policies and practices to minimize further the privacy risks posed by their operations (12, 18, 19, 27–29).

This includes utilizing the “separation principle” (30), a simple method for restricting the type of data received by each organization in the linkage process. Under this principle, the DLU receives only the personally identifying information required for linkage, but not the content data. The researcher, on the other hand, receives only the content but not personal identifying information. Only the data custodian has access to both personal identifying information and clinical content data.

The use of the separation principle greatly enhances privacy. However, in many instances, the risk to privacy can be still large. For instance, knowledge that a particular individual has a record within a data collection is itself revealing, especially for specific data collections such as mental health inpatient datasets or cancer registries. This information will be still provided to the linkage unit under the separation principle.

The release of personally identifying information always carries some additional risk, as more individuals have access to this information. While rare, attempting to determine whether a person of interest is contained within a dataset does occur; for instance, US intelligence agents have used their surveillance capabilities to spy on romantic interests (31), as have Australian telecommunications workers (32).

Some custodians remain averse to the release of personal information for reasons that extend beyond privacy risks, such as discrimination, reputational damage and/or embarrassment, criminal misuse of the data, and commercial harm (25).

Legislative barriers and risk aversion by data custodians are currently being challenged by open data policies and a growing need by and for government to work with private industry to more effectively service community needs. A recent Productivity Commission Inquiry into the benefits and costs of increasing the availability and use of public and private sector data recognizes the barriers and risks associated with working with named data (25). The Inquiry outlines a framework for data sharing underpinned by legislative change, governance structures (to remove blocks and increase data access), and the development of “systems and processes […] to identify, assess, manage and mitigate risks related not just to data release and sharing, but also data collection and storage” [(25), p.9].

The issues being encountered in Australia are shared internationally. DLUs in the United States, Canada, and Europe face similar legal and risk-related hurdles (e.g., the United States: *Health Insurance Portability and Accountability Act 1996*, Canada: *Personal Health Information Protection Act 2004*, and Europe: *Data Protection Directive 95/46/EC*). German laws in relation to the disclosure of personal information are particularly restrictive (*Bundesdatenschutzgesetz—Federal Data Protection Act of Germany*) and, in some cases, only a single data item can be used for anonymous linkage (33).

## Privacy-Preserving Solutions

Privacy-preserving record linkage protocols utilize algorithms and techniques to conduct linkage on encrypted or masked information; these methods do not require data custodians to release personal identifiers to third parties. This reduces the risks associated with the release of personal data. Three important attributes characterize all PPRL protocols: accuracy, efficiency, and privacy.

Different classes of privacy-preserving linkage methods provide differing levels of privacy protection. These range from techniques such as the statistical linkage key that simply amalgamates parts of a person’s identifiers into a single variable (34) to methods that encrypt or encode the data so that those with access cannot learn any information directly from the encrypted values. The exact level of privacy required will always depend on context, but all things being equal, a protocol with higher privacy is preferred.

An important difference in PPRL protocols is the method of matching which impacts on linkage quality (accuracy). Protocols may perform matching on a particular set of identifiers, using either exact or similarity comparisons. Similarity matching enables records with slight differences to come together, which is vital for obtaining high-quality linkage results (accuracy). For this reason, PPRL protocols that utilize approximate matching are favored.

Efficiency can be often a concern for record linkage and will continue to present challenges to DLUs as the volume of data continues to grow. Although there are no established performance standards, record linkage is computationally slow, and for any PPRL protocol to be practical, it must complete within a reasonable time frame.

The extent to which these protocols are used in practice varies. To date, most PPRL implementations use exact matching on particular attributes of a dataset (35), which are typically irreversibly encoded to ensure privacy (36). Though efficient, these methods have reduced linkage quality and, therefore, are operationally unsuitable in DLUs.

Of all PPRL methods, the Bloom filter method appears to be the most promising for operational use (37). An advantage of the Bloom method over other PPRL methods is that it utilizes approximate matching while providing similar or superior privacy protection. The method has been evaluated on large-scale, real world health datasets, with results returning equal linkage quality and similar efficiency to traditional linkage methods (which use personal identifiers in the matching process) (38). No record linkage method, privacy preserving or not, achieves perfect accuracy—to be able to achieve equal accuracy to the standard non-privacy-preserving approach is a considerable accomplishment. The security of the protocol has been rigorously investigated (39–41). Cryptographic attacks on the algorithm found ways to reveal some identifiers (40). However, modifications to the protocol have rendered these attacks fruitless (42); there are currently no known security vulnerabilities with the protocol.

The introduction of the Bloom filter method brings new challenges (17). As well as operational requirements around designing optimal linkage strategies, new ways of validating record linkage results need to be developed. In traditional record linkage, linkage results are validated through clerical inspection (or “manual review”) of personal identifiers; however, in a privacy-preserved context where all data are encoded, there is no way to manually review the data or correct possible data or linkage errors. New methods for validating linkage results under privacy-preserved linkage model are emerging, however (43).

## PPRL: An Example

Consider the (hypothetical) scenario: to attempt to reduce the rate of youth suicide, the government of the day has invested in a comprehensive mental health care package for those who have attempted suicide. The government wishes to see whether their program has worked in reducing the rate of suicide and attempted suicide.

To answer this question, two datasets will be required: a hospital admissions dataset and a mortality register. From the hospital admissions dataset, records will be required to be sent to the linkage unit for all those persons who have attempted suicide before and after the start of the health intervention; all records from the mortality register will be required by the linkage unit. The linkage unit will receive only the personal identifying information required for linkage (i.e., name, date of birth, gender, address). The linkage unit identifies which records from the supplied hospital dataset have associated mortality records. The linkage unit passes this information back to the data custodians, who then provide the content data (i.e., not personally identifying information) to the researcher for the hospital records, and any linked mortality records, along with a key that identifies which records belong to which individual. The researcher can then use this information to determine whether the intervention reduced suicide and attempted suicide rates.

The privacy risk in the aforementioned scenario is the delivery to the linkage unit of personal identifying information from hospital records of those who have attempted suicide. This extremely sensitive information has been made available to a third party. The use of privacy-preserving linkage methods would remove this risk; instead, the linkage unit would receive encrypted personal identifiers; they would have no means of identifying any of these individuals, but would still have the ability to determine which records belong to the same individual between datasets.

## Growing International Interest in PPRL

With a growing demand for linked data from government and the university sector, interest in PPRL, particularly the Bloom filter method, is flourishing. Interest stems from two principal sources: at a technical level, by computer scientists and cryptographers with interests in information and data security, and at an operational level, by groups with interest in and responsibility for delivering record linkage services.

Several groups are actively developing and refining PPRL methods at the scientific level including the German Record Linkage Center (University of Duisburg-Essen) (44, 45), the Research School of Computer Science (Australian National University) (46–48), and the Health Information Privacy Laboratory (Vanderbilt University) (39, 49). Researchers from these groups and others recently participated in a 2016 Data Linkage and Anonymisation programme at the Isaac Newton Institute for Mathematical Sciences (Cambridge University, supported by EPSRC grant no EP/K032208/1)^2^; this 6-month international programme included seminars and workshops on linkage and privacy protection to share and advance knowledge in the mathematical sciences and related disciplines. A key goal of the forum was to “enhance opportunities for the analysis of data, especially obtained through linkage, whilst protecting privacy and taking account of related practical constraints.”

At an operational level, PPRL featured prominently in the 2016 International Population Data Linkage Network Conference (Swansea University), with several presentations on the topic including a keynote session that described a collaboration between international research institutions in Canada, Australia, and Wales (44, 46, 50–53).

## Opportunity and Change Management

In addition to reducing the privacy risks associated with record linkage, the advent of PPRL protocols potentially heralds a new era of population-focused research using linked data, bridging gaps, and opening up opportunities for new and different forms of linkage-based research. PPRL methods may provide an avenue to access previously “hard to get” datasets (i.e., those with significant legal or regulatory constraints). PPRL methods may also provide a mechanism for accessing and integrating data from new and emerging sources. As well as data from new technologies (e.g., wearable devices, smartphone apps), these new sources may include the private health sector that has, to date, had limited exposure to, and engagement with, data linkage frameworks (54, 55).

New methods may require new or adjusted models of operation. Some custodians have expressed a desire to have flexibility in record linkage models to accommodate the features of different data collections (50). However, different or altered data linkage operating models can have significant implications for end-user timeframes, operational efficiency, and linkage quality (50), and these need to be carefully managed and monitored. It is important that the strengths and limitations of the PPRL methods are understood. This will require conversations with stakeholders (i.e., data custodians, linkage units, researchers, and the community) around the risk–benefit of these new models and the expected realization of public benefit.

## Conclusion

The implementation of PPRL methods that do not require the release of personal information but protect privacy through other mechanisms (e.g., encryption methods) represents a breakthrough in record linkage, substantially reducing privacy risks without negatively impacting on linkage quality. By utilizing methods that do not require the release of personally identifying information, concerns regarding personal surveillance and government overreach can be allayed. Supplementing traditional linkage methods with PPRL methods will increase the number and type of datasets that can be included in record linkage studies.

The advent of PPRL methods to protect patient privacy expands the toolkit of techniques that are available to DLUs. Used in conjunction with traditional linkage methods, PPRL widens the net of record linkage without compromising privacy or linkage quality. These methods will hopefully allow more diverse, patient-centered data sources to be utilized for health research, bringing enormous opportunities to increase our understanding of disease and to tailor interventions and treatment to each individual.

## Author Contributions

AB and AF accept immediate responsibility for the manuscript. AF, AB, SR, JB, and JS each contributed to the conception and design of the paper. AF and AB drafted the first version of the article, with SR, JB, and JS providing important additional input and intellectual content. All authors were involved in revising the manuscript and approving its final form.

## Conflict of Interest Statement

The authors declare that the research was conducted in the absence of any commercial or financial relationships that could be construed as a potential conflict of interest.

## Footnotes

^1^In Western Australia, for example, both the WA Children’s Court Act 1988 and the Young Offenders Act 1994 curtail the release of information for research in relation to juvenile offenders. In South Australia, state-based regulations restrict the release of information from the SA Perinatal Statistics Collection (SA Health Care Variation Regulations 2010, Reg 4). Similar legal barriers exist in other jurisdictions, both locally and internationally.

^2^https://www.newton.ac.uk/event/dla.
